# Poor Reliability between Cochrane Reviewers and Blinded External Reviewers When Applying the Cochrane Risk of Bias Tool in Physical Therapy Trials

**DOI:** 10.1371/journal.pone.0096920

**Published:** 2014-05-13

**Authors:** Susan Armijo-Olivo, Maria Ospina, Bruno R. da Costa, Matthias Egger, Humam Saltaji, Jorge Fuentes, Christine Ha, Greta G. Cummings

**Affiliations:** 1 CLEAR (Connecting Leadership and Research) Outcomes Research Program, Faculty of Nursing, University of Alberta, Edmonton, Alberta, Canada; 2 Faculty of Rehabilitation Medicine, Department of Physical Therapy, University of Alberta, Edmonton, Alberta, Canada; 3 Emergency Medicine Strategic Clinical Network, Alberta Health Services, Department of Emergency Medicine, Faculty of Medicine & Dentistry, University of Alberta, Edmonton, Alberta, Canadá; 4 Department of Physical Therapy, Florida International University, Miami, Florida, United States of America; 5 Institute of Social & Preventive Medicine, University of Bern, Bern, Switzerland; 6 Orthodontic Graduate Program, School of Dentistry, University of Alberta, Edmonton, Alberta, Canadá; 7 Faculty of Rehabilitation Medicine, University of Alberta, Edmonton, Alberta, Canada; 8 Catholic University of Maule, Department of Physical Therapy, Talca, Maule, Chile; 9 Rehabilitation Research Center, Faculty of Rehabilitation Medicine, University of Alberta, Edmonton, Alberta, Canada; University of Chieti, Italy

## Abstract

**Objectives:**

To test the inter-rater reliability of the RoB tool applied to Physical Therapy (PT) trials by comparing ratings from Cochrane review authors with those of blinded external reviewers.

**Methods:**

Randomized controlled trials (RCTs) in PT were identified by searching the Cochrane Database of Systematic Reviews for meta-analysis of PT interventions. RoB assessments were conducted independently by 2 reviewers blinded to the RoB ratings reported in the Cochrane reviews. Data on RoB assessments from Cochrane reviews and other characteristics of reviews and trials were extracted. Consensus assessments between the two reviewers were then compared with the RoB ratings from the Cochrane reviews. Agreement between Cochrane and blinded external reviewers was assessed using weighted kappa (κ).

**Results:**

In total, 109 trials included in 17 Cochrane reviews were assessed. Inter-rater reliability on the overall RoB assessment between Cochrane review authors and blinded external reviewers was poor (κ  =  0.02, 95%CI: −0.06, 0.06]). Inter-rater reliability on individual domains of the RoB tool was poor (median κ  = 0.19), ranging from κ  =  −0.04 (“Other bias”) to κ  =  0.62 (“Sequence generation”). There was also no agreement (κ  =  −0.29, 95%CI: −0.81, 0.35]) in the overall RoB assessment at the meta-analysis level.

**Conclusions:**

Risk of bias assessments of RCTs using the RoB tool are not consistent across different research groups. Poor agreement was not only demonstrated at the trial level but also at the meta-analysis level. Results have implications for decision making since different recommendations can be reached depending on the group analyzing the evidence. Improved guidelines to consistently apply the RoB tool and revisions to the tool for different health areas are needed.

## Introduction

The term “quality assessment” has been used extensively in the literature, particularly in the context of systematic reviews, to refer to the critical appraisal of primary studies. Different approaches to quality assessment have been proposed for assessing the quality of studies [1], [2]. A variety of methods (scales and checklists) have been used by different Cochrane Review groups [3], [4]; however, because of methodological inconsistencies across quality instruments and the lack of empirical evidence supporting their validity and reliability [5], [6], the use of these methods was explicitly discouraged in Cochrane reviews [3].

In 2008, the Cochrane Collaboration (CC) initiated a shift in the approach to the evaluation of trial quality by linking the concept of quality to the internal validity of a study (risk of bias; the extent to which the design and conduct of a study are likely to prevent bias) [3]. The Cochrane Collaboration developed the Risk of Bias tool (RoB) as a method to assess risk of bias based on study design and conduct rather than relying on general reporting issues of trial characteristics [3]. Since then, the Cochrane Collaboration has required the use of the RoB tool to establish consistency in the assessment of study quality across Cochrane Review groups.

The RoB tool is based on six domains and 7 items: sequence generation, allocation concealment, blinding, incomplete outcome data, selective outcome reporting, and “other sources of bias.” Critical assessments of the risk of bias (high, low, unclear) in each domain are made separately for each outcome in a given study. The choice of these components for inclusion in the tool was based on empirical evidence of their association with effect estimates [5], [7], [8]; Recent research [9], [10] recommends further testing of the psychometric properties (i.e., validity, reliability, and responsiveness) of the RoB tool, and evaluations of the tool in a broad range of research fields. In addition, researchers have called for the use of clear and consistent guidelines and classification systems to apply and interpret the RoB tool [11]. This information is essential since differences in the appraisal and interpretation of risk of bias across trials can explain variation in the interpretation of results of studies included in a systematic review, and ultimately impact the conclusions and clinical practice.

Despite the RoB tool being increasingly used in Cochrane reviews; few studies have assessed its psychometric properties, specifically in paediatric trials, general medical and oncology trials [9], [10], [12], [13]. Ihe inter-rater agreement for the individual domains of the RoB tool has been found to range from poor (κ [kappa]  =  0.13 for selective reporting) to substantial (κ  =  0.74 for sequence generation) [9]. A recent study [13] assessed the reliability of the RoB tool between individual reviewers and across consensus ratings of pairs of reviewers on a sample of 154 and 30 randomized clinical trials (RCTs) published in the general medical literature respectively. The study found that the reliability between pairs of reviewers was “fair” for most of RoB domains with kappa values ranging from 0.2 to 0.34. However, the agreement between consensus ratings was always poorer than the agreement between pairs of reviewers indicating a high variability in interpreting and applying the RoB tool across different systematic review groups and across systematic reviews [13]. This agreement in consensus ratings (across pair of reviewers) was conducted only on 30 trials within a group of reviewers from the same team using guidelines developed specifically for the study.

The reliability of the RoB tool has not been investigated by comparing ratings of an external blinded panel of reviewers with those obtained from authors of Cochrane reviews. This work is of crucial importance for researchers who incorporate risk of bias assessments from Cochrane- and non-Cochrane systematic reviews into meta-epidemiological research approaches, since risk of bias assessments obtained by different research group can lead to different results. Furthermore, the reliability of the RoB in the context of physical therapy (PT) trials has not yet been evaluated. The objectives of this study were to test the inter-rater reliability of the RoB tool applied to PT trials by comparing consensus ratings from Cochrane review authors with those of blinded external reviewers, and to investigate potential sources of disagreements to inform the use of the RoB tool.

## Methods

The Cochrane Database of Systematic Reviews (CDSR) was systematically searched from 2005 to May 25 2011 for meta-analyses of PT interventions using the words physical therapy, physiotherapy, rehabilitation, exercise, electrophysical agents, acupuncture, massage, transcutaneous electrical stimulation (TENS), interferential current, ultrasound, stretching, chest therapy, pulmonary rehabilitation, manipulative therapy, mobilization, and related terms. For a detailed search strategy see **Appendix S1**. Meta-analyses and their RCTs were included if: 1) the meta-analysis included at least 5 RCTs, with at least one of the interventions being currently or potentially part of PT practice according to the World Confederation for Physical Therapy (WCPT) [14]; 2) the outcome of interest in the meta-analysis (explicitly described as the main outcome or the outcome with the largest number of trials) was continuous; and 3) the RoB tool was used for assessment of individual trials. A unique identifier was assigned to meta-analyses and trials that met the inclusion criteria.

### RoB assessments procedure

The risk of bias of individual trials included in the meta-analyses was assessed on 6 domains (7 items) of the RoB tool [15]: sequence generation, allocation concealment, blinding of participants and personnel, blinding of outcome assessors, incomplete outcome data, selective outcome reporting, and other sources of bias. We followed the guidelines established by the Cochrane Collaboration to perform RoB assessments; however we developed specific decision rules to make decisions **(Appendix S2)**. Risk of bias evaluations for blinding and incomplete outcome data were based upon the primary (continuous) outcome of interest selected for meta-analysis in the Cochrane review. If not clearly specified, the outcome was chosen according to the meta-analysis that contained the largest number of trials in the review. The Cochrane guidelines recommend using trial protocols to complete assessments of selective outcome reporting bias. However, due to the low likelihood of locating protocols for trials, we did not search for study protocols [24]. Therefore, for the category of “low” risk of bias, it was required that trial publications reported all primary and secondary outcomes in the methods and results sections, with new outcomes not being added in the results section. If the primary outcome of the trial was not included in the results, there was a high risk of selective outcome reporting bias. In addition we paired outcomes reported in methods and results sections. If more than 70% of the secondary outcomes were not reported in the results or methods sections, then the study was rated as high RoB. For ‘other bias’, we looked at baseline comparability, control for co-interventions (contamination bias) and whether treatment compliance was acceptable. These criteria have been used in the risk of bias assessments of the Cochrane Back Review Group to determine other sources of potential bias [16].

For the overall assessment of RoB, a trial was considered at low risk of bias if it was rated as low risk in all individual domains; if the rating was unclear in at least one domain, and the other domains were unclear or low, the overall assessment of RoB was unclear. Finally, an overall assessment of high risk of bias was considered if at least one domain was rated as high [12], [13].

Two independent reviewers (**any of these reviewers: SAO, JF, HS, CH, AC, DP**) blinded to the RoB ratings reported in the Cochrane reviews assessed the risk of bias of all PT trials included in the meta-analyses. **Each pair of reviewers assessed risk of bias in each study and disagreements were resolved by discussion between reviewers until consensus was reached**. If consensus was not achieved, a final decision on RoB assessments was reached after consultation with a third reviewer (first author), **although this was not necessary**. Blinding of the external panel of reviewers was achieved as follows: 1) reviewers were not told the objective of this study; 2) they were not provided with RoB assessments performed by Cochrane reviewers; 3) after the external panel of reviewers completed their assessments, an independent reviewer who was not part of the review panel extracted RoB data assessment performed by Cochrane reviewers **(MO)**. The integrity of blinding was assessed by asking the reviewers post hoc if they had checked the Cochrane RoB assessment. None of them reported that they did.

Data on RoB assessments from Cochrane reviews and other characteristics of reviews and trials were extracted by one reviewer (MO or SAO) and entered directly into a pilot tested electronic form. Consensus assessments between the two reviewers **from our panel** were then compared with the RoB ratings from the Cochrane reviews. In addition, two reviewers independently assessed the RoB at the meta-analysis level for both groups of reviewers (i.e. external panel of reviewers and Cochrane reviewers) using the guidelines established by the Cochrane handbook [15], [17]. A low, unclear and high RoB at the meta-analysis level was defined as: “most information is from studies at low, unclear or high risk of bias respectively” [15], [17]. Since no further guidance is in the Cochrane handbook, we established an arbitrary cut-off value of 60% to define the “majority of studies”. Assessments were compared and discrepancies were resolved by consensus between reviewers.

### Characteristics of the reviewers' panel

Six reviewers with experience in different areas of health sciences research comprised the review panel in this study. Two reviewers had a Bachelor in Health Sciences **(CH, AC)**, one had a Masters in Public Health **(DP)**, one had a Masters in Dentistry and currently working on a PhD in Orthodontics **(HS)**, and two were physical therapists and had Masters and PhD in Rehabilitation sciences **(SAO, JF)** with at least 10 years of experience in critical appraisal and systematic reviews. Four of them **(DP, HS, SAO, and JF)**, had formal training in critical appraisal and systematic reviews. The other 2 **(CH, AC)** had at least one year of hands-on experience conducting systematic reviews. **Four of the reviewers (SAO, JF, HS, CH) were part of the research team collaborating in this project and two of them (DP, AC) were hired to perform the data extraction and quality assessments. All of them verbally agreed to participate as reviewers in this study.**

### Training process

All reviewers were trained and received guidelines for RoB assessments from the first author **(SAO)** who was a physical therapist by training and had a MSc and PhD in Rehabilitation Sciences and more than 10 years of experience in critical appraisal and systematic reviews. Reviewer training was carried out using 10 trials not included in the study. Results of RoB assessments for these 10 studies were independently reviewed and discussed in a group meeting to determine consistency in ratings. In addition, the team members met on a regular basis to further calibrate RoB assessments throughout the study.

### Statistical analysis

Inter-rater reliability of RoB assessments between Cochrane and blinded external reviewers [18]–[20] and within the panel of external reviewers was assessed using weighted kappa (κ) for categorical data. Inter-rater scores for both individual domains and overall assessments of the RoB tool were considered. Analyses were conducted using STATA (version 12, Stata Corp; College Station, Texas; USA). For raw data for each domain see **Appendix S3**.

Criteria proposed by Byrt [21] were used to interpret kappa values. Values between 0.93–1.00 represented excellent agreement; 0.81–0.92 very good agreement; 0.61–0.80 good agreement; 0.41–0.60 fair agreement; 0.21–0.40 slight agreement, 0.01–0.20 poor agreement; and 0.00 or less were considered to have no agreement.

## Results

### Literature search

**Figure 1:**
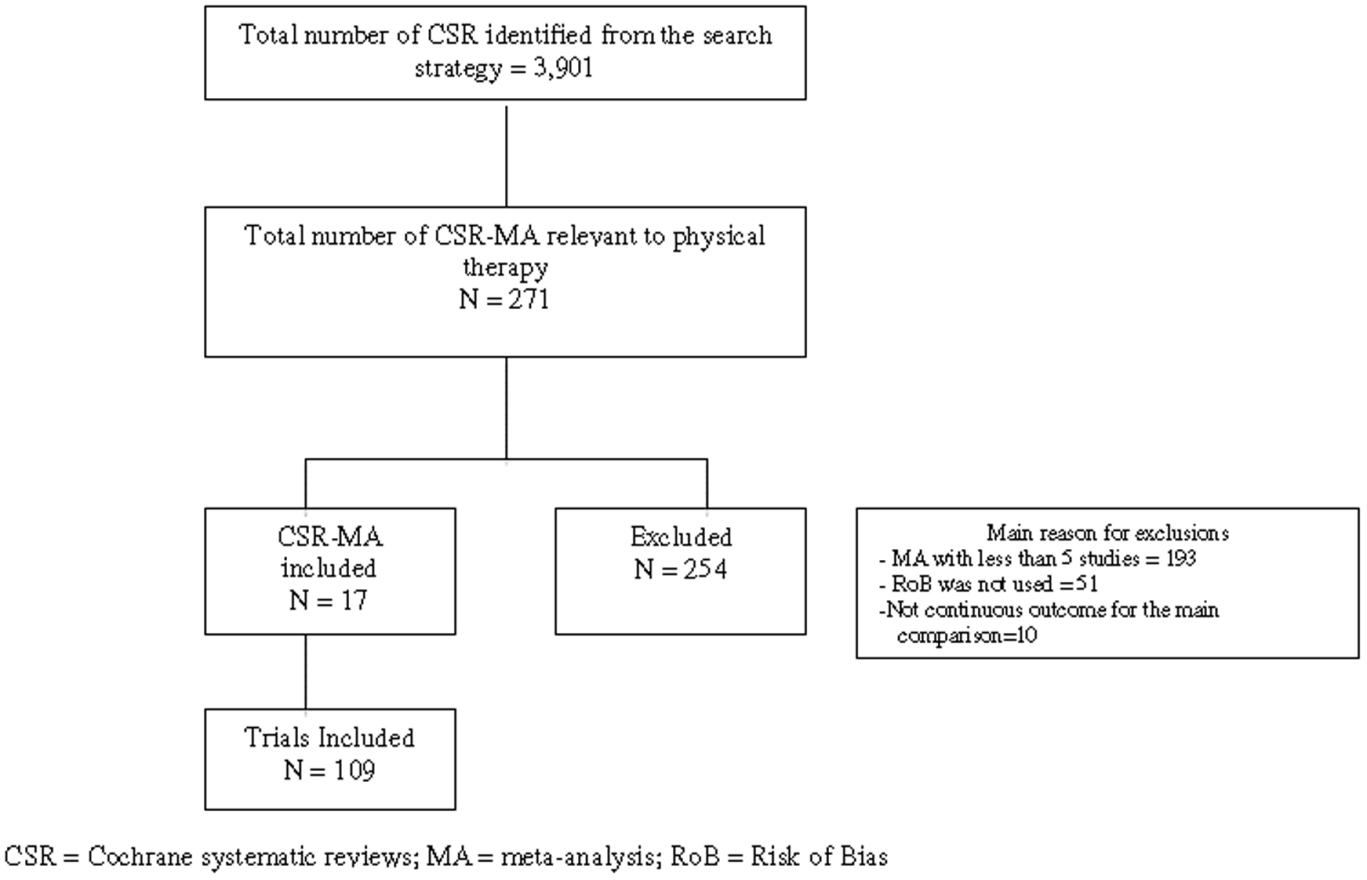
Diagram for the identification of reviews.

The systematic search of the CDSR resulted in the identification of 3901 Cochrane review titles, with 271 reviews being potentially relevant to physical therapy. Of these, 68 Cochrane reviews included a meta-analysis of at least five studies on PT interventions assessing a continuous outcome. Figure 1 outlines the retrieval of Cochrane reviews and the number of trials included in the analysis. A total of 109 trials included in 17 Cochrane reviews that used the RoB tool were assessed. Table 1 summarizes the characteristics of the Cochrane reviews included in the study.

**Table 1:** Characteristics of Cochrane systematic reviews on physical therapy interventions that provided trial data for the analysis of inter-rater reliability of RoB.

Author	Review title	Area	Number of RCTs	Outcome Reported for Quality assessment	Outcome selected for External Panel Assessments	Number of RoB assessors in Cochrane Review	Interrater-reliability in Cochrane review
Effing et al., 2009 [33]	Self-management education for patients with chronic obstructive pulmonary disease	Cardiorespiratory PT	6	Not specified	HRQoL	2	Not reported
Sirtori et al, 2009 [36]	Constraint-induced movement therapy for upper extremities in stroke patients	Neurology PT	6	All outcomes	Disability post-intervention	2	Not reported
Taylor et al. 2010 [35]	Home-based versus centre-based cardiac rehabilitation	Cardiorespiratory PT	11	All outcomes	Exercise capacity	1	Not reported
Rutjes et al, 2010 [30]	Transcutaneous electrostimulation for osteoarthritis of the knee	Musculoskeletal PT	12	Pain and Function	Pain	2	Not reported
Orozco et al, 2008 [39]	Exercise or exercise and diet for preventing type 2 diabetes mellitus	General PT (Chronic conditions)	7	All outcomes	Fasting plasma glucose (mml/L)	2	Not reported
Kramer et al, 2010 [38]	Aerobic exercise for women during pregnancy	Gynaecological PT	6	All outcomes	Birth weight	1	Not reported
Harvey et al, 2010 [25]	Continuous passive motion following total knee arthroplasty in people with arthritis	Musculoskeletal PT	5	Not specified	Active knee ROM	2	Not reported
Rutjes et al, 2010 [29]	Therapeutic ultrasound for osteoarthritis of the knee or hip	Musculoskeletal PT	4	Pain and Function	Pain	2	Not reported
Handoll et al, 2009 [24]	Multidisciplinary rehabilitation for older people with hip fractures	Musculoskeletal PT	7	Function and HRQoL	HRQoL	2	Not reported
Katalinic et al, 2010 [26]	Stretch for the treatment and prevention of contractures	Musculoskeletal PT	7	Not specified	Joint mobility	2	Not reported
Davies et al, 2010 [54]	Exercise based rehabilitation for heart failure	Cardiorespiratory PT	9	All outcomes	HRQoL	1	Not reported
Manheimer et al, 2010 [27]	Acupuncture for peripheral joint osteoarthritis	Musculoskeletal PT	8	Pain and Function (WOMAC)	Pain	2	Not reported
Schaafsma et al, 2011 [31]	Physical conditioning programs for improving work outcomes in workers with back pain	Musculoskeletal PT	5	All outcomes	Time to return to work	2	Between the 2 assessors, there was an average of one or two items of disagreement for every study.
Ostelo et al, 2011 [28]	Rehabilitation after lumbar disc surgery	Musculoskeletal PT	3	All Outcomes	Pain	2	Not reported
Fransen M., et al., 2009 [23]	Exercise for osteoarthritis of the hip	Musculoskeletal PT	5	All outcomes	Pain	3	Not reported
Puhan MA et al, 2010 [34]	Pulmonary rehabilitation following exacerbations of chronic obstructive pulmonary disease	Cardiorespiratory PT	5	HRQoL, Hospital Admission And Walk test	HRQoL,	2	Not reported
States et al., 2009 [37]	Overground physical therapy gait training for chronic stroke patients with mobility deficits	Neurology PT	6	Not specified	Gait speed	3	The authors demonstrated 100% agreement on the Cochrane items.

HRQoL  =  Health-related quality of life; PT = physical therapy; RCT  =  randomized controlled trial; RoB  =  Risk of Bias; ROM  =  range of motion; WOMAC  =  Western Ontario and McMaster Universities Arthritis Index.

### Characteristics of selected studies

Briefly, the reviews were published between 2008 and 2011 and included meta-analyses of the effectiveness of PT interventions for musculoskeletal (9 reviews [22]–[30] cardiorespiratory (4 reviews) [31]–[34], neurological (2 reviews) [35], [36], gynaecological (1 review) [37], and general conditions (1 review) [38].

The majority of Cochrane reviews (15 reviews) did not include a formal evaluation of the inter-rater reliability of the RoB assessments. Although the majority of reviews stated that two independent reviewers assessed study RoB, in four reviews, a single reviewer assessed RoB, with verification by a second reviewer. Similarly, twelve of the 17 (71%) Cochrane systematic reviews did not clearly specify the outcome used for the RoB assessments, whereas eight out of 17 (47%) of systematic reviews combined all outcomes into a single bias assessment.

A median number of six trials were included in the meta-analyses (interquartile range: 5, 8). All but one cross-over trial were identified as parallel trials. The majority of trials (n  =  93) used active controls whereas 15 trials were placebo-controlled. The control group of one trial was not clearly identified. Seventy-five trials were efficacy trials; 26 effectiveness trials, and seven trials combined an evaluation of the efficacy/effectiveness of PT interventions. One trial was not clearly described as an efficacy or effectiveness trial.

The number of trials available for assessing the inter-rater reliability of both individual-domain and overall RoB assessments varied as not all Cochrane reviews reported ratings for all the domains of the RoB tool. Inter-rater reliability of RoB assessments between Cochrane review authors and blinded external reviewers and the inter-rater reliability within the external panel of reviewers are presented in Table 2.

**Table 2:** Reliability between Cochrane Reviewers and External Panel and Reliability for the External Panel.

		Reliability External Panel vs. Cochrane Reviewers			Within External Panel Reliability		
Domain	N trials included in Analysis	Kappa	95%CI	Classification	Kappa	95%CI	Classification
Sequence generation	109	0.62	0.46, 0.76	good agreement	0.71	0.58, 0.84	good agreement
Allocation concealment	108	0.30	0.12, 0.43	slight agreement	0.79	0.65, 0.93	good agreement
Blinding of participants and personnel	86	0.15	0.04, 0.24	poor agreement	0.56	0.54, 0.59	fair agreement
Blinding outcome assessment	97	0.41	0.37, 0.48	slight agreement	0.54	0.36, 0.62	fair agreement
Incomplete data	96	0.24	0.07, 0.32	slight agreement	0.71	0.69, 0.73	good agreement
Selective reporting	87	0.13	0.05, 0.32	poor agreement	0.50	0.29, 0.63	fair agreement
Other bias	78	−0.04	−0.08, 0.04	poor agreement	0.32	0.25,0.42	fair agreement
Overall rating	108	0.02	−0.06, 0.06	poor agreement	0.55	0.40, 0.70	fair agreement

### Inter-rater agreement: Cochrane review authors vs. blinded external reviewers

Inter-rater reliability on the overall RoB assessment between Cochrane review authors and blinded external reviewers was poor (κ  =  0.02, 95%CI: −0.06, 0.06). Inter-rater reliability on individual domains of the RoB tool was poor (median κ  = 0.19), ranging from κ  =  −0.04 (“Other bias”) to κ  =  0.62 (“Sequence generation”). Table 2 displays the inter-rater reliability of the RoB tool between the blinded external review panel versus Cochrane reviewers.

When overall RoB categories assigned by blinded external reviewers were compared to those of Cochrane review authors, we found that the number of trials assessed as “low” risk of bias by Cochrane review authors (n  =  9) was greater than blinded external reviewers (n  =  3). Similarly, the number of trials rated as “high” risk of bias by Cochrane review authors (n  =  66) was greater than blinded external reviewers (n  =  31). In contrast, blinded external reviewers had a greater number of trials assessed as “unclear” in the overall RoB assessment (n  =  74) compared to Cochrane review authors (n  =  33). The main source of disagreement between Cochrane review authors and blinded external reviewers in the overall rating of RoB was due to discrepancies in the classification of “unclear” vs. “high” risk of bias; with 45 trials rated as “high” risk of bias by Cochrane review authors and “unclear” by blinded external reviewers.

### Inter-rater agreement within the panel of blinded external reviewers

The inter-rater reliability between blinded external reviewers on the overall RoB rating was fair (κ  =  0.55, 95%CI: 0.40, 0.70). Inter-rater reliability on individual domains of the RoB tool was fair (median κ  = 0.56) ranging from κ  =  0.32 (“Other bias”) to κ  =  0.79 (“allocation concealment”).

### Overall RoB at the Meta-analysis level

There was no agreement (κ  =  −0.29, 95%CI: −0.81, 0.35) in the overall RoB assessment at the meta-analysis level between Cochrane review authors and blinded external reviewers. Cochrane reviewers had evaluated 10 meta-analyses as high RoB while the external panel of reviewers classified them as “unclear”. **Table 3** displays the RoB assessment at the meta-analysis level.

**Table 3:** Comparison of Overall ratings at the meta-analysis level between external panel and Cochrane reviewers.

Meta-Analysis	Overall RoB meta-analysis level External Panel reviewers	Overall RoB meta-analysis level Cochrane reviewers
**Agreements**		
Orozco, 2008	unclear	unclear
Sirtory, 2009	unclear	unclear
Davies, 2010	unclear	unclear
**Disagreements**		
States, 2009	unclear	high
Fransen, 2009	high	low
Handoll, 2009	high	unclear
Effing, 2009	high	unclear
Taylor, 2010	unclear	high
Harvey, 2010	unclear	high
Rutjes, 2010	unclear	high
Katalinic, 2010	unclear	high
Puhan, 2010	high	high
Kramer, 2010	unclear	high
Rutjes, 2010b	unclear	high
Manheimer, 2010	unclear	high
Ostelo, 2011	unclear	high
Schaafsma, 2011	unclear	high

## Discussion

Based on the assessment of RCTs included in Cochrane reviews of PT interventions, this study found that the inter-rater reliability of RoB assessments between Cochrane review authors and blinded external reviewers was poor. This result confirms the findings of previous studies regarding the poor reliability of the RoB tool domains in other areas of health research [9], [10], [12], [13]. Our results indicated that RoB assessments in Cochrane reviews could not be replicated consistently by an external panel of reviewers using consensus RoB assessments.

Consensus ratings are of crucial importance since they are commonly used in systematic reviews. Only one previous study assessed the reliability of the RoB based on consensus assessments across pairs of reviewers from four research centres using a sample of 30 trials indexed in PubMed between 2000 and 2006 [13]. Using a larger number of trials in PT and comparing the RoB consensus ratings between blinded external reviewers and Cochrane reviewers, our study confirmed that agreement across pairs of reviewers is generally lower than agreement between reviewers. Cochrane reviews have long been considered the gold standard for systematic reviews in health care. Results of our study have important implications for the interpretation of results of RoB assessments across Cochrane reviews and produced by different Cochrane Review Groups. The poor agreement in RoB assessments between Cochrane reviewers and an external panel of reviewers has raised several concerns: 1) RoB assessments cannot be reproduced by different groups of reviewers. If true, it would mean that RoB assessments are not reliable and depend on the reviewers' level of knowledge and familiarity with the information provided in the individual trials; 2) the RoB tool is a very subjective tool that cannot provide reliable assessments; 3) despite efforts by the Cochrane Collaboration to establish high quality standards for conducting systematic reviews, poor agreement appears to be the norm rather than the exception when conducting RoB assessments. Thus, we pose the following questions: can we trust risk of bias results reported in Cochrane reviews? Can we trust assessments using the RoB tool?

The low reliability of RoB assessments between our panel of blinded external reviewers and Cochrane reviewers has implications for researchers who use bias ratings from Cochrane reviews or other external sources to conduct meta-epidemiological research on the relationship between trial characteristics and over and under-estimation of treatment effects, since bias ratings obtained by different research group can lead to different results. For example, authors of meta-epidemiological studies [8], [39], [40], have taken information from external sources (Cochrane assessments, or information provided by authors of reviews). Although using data reported in the reviews, it is a practical and cost-efficient way to obtain information, authors should be aware that these evaluations may be inconsistent and prone to bias due to many factors such as expertise, training, level of education, and other characteristics of reviewers making quality judgements.

Very low agreements among Cochrane reviewers and the external panel were obtained for allocation concealment, blinding of participants, blinding of outcome assessment, and incomplete data. These features of a trial can have a substantial impact on the estimates of treatment effect [5], [9], [40]–[42]. **Some studies, for example, have found that** inadequate allocation concealment or lack of double-blinding can overestimate treatment effects on average by 18% and 9%, respectively [5], [40], [42]. **Nevertheless, other studies have found that trials with adequate allocation concealment and blinding had higher treatment effects than trials that did not accomplish with these methodological features.** [43], [44] Similarly, effect sizes from trials that excluded dropouts in the analysis or considered a modified intention to treat (ITT) approach were more likely to show a beneficial effect than trials without exclusions, demonstrating that the ITT principle is important to preserve the benefits of randomization and keep unbiased estimates [45]–[47]. Over-estimates of treatment effects, or bias, at the trial level, can lead to biased or inaccurate results and conclusions in systematic reviews and meta-analyses [40], [41], [48]–[50]. In addition, our analyses showed no agreement between decisions made based on RoB assessments at the level of meta-analysis. This means that both groups of reviewers did not agree in the overall quality of the evidence at the meta-analysis level. These factors can ultimately have repercussions on decision-making and quality of patient care since different assessments could lead to different decisions for clinical practice. Therefore, is alarming that the disagreements obtained between the two panels of reviewers are worse when it matters most.

The selection of different outcomes for RoB assessments may have influenced the poor agreement between Cochrane reviewers and a panel of blinded external reviewers. The majority of Cochrane reviews analyzed did not clearly specify the outcome used for RoB assessments. This directly reduces reproducibility of RoB assessment for outcome-dependent domains of the tool. Cochrane reviewers should report RoB assessments separately for each outcome analyzed, or at least for the main outcomes of the review. Half of the systematic reviews included in this study combined all outcomes into a single bias assessment and therefore, it is uncertain for which outcome the RoB assessments were applicable. Cochrane reviewers should clearly state which outcomes were used to perform the RoB assessments, in order to allow reproducibility and comparison.

The RoB has been extensively used by many Cochrane reviews, albeit the information of the inter-rater reliability of RoB is rather limited. To date, five studies [9], [10], [12], [13], [51] have investigated the inter-rater reliability of the RoB. One of them [51] did not use the generic RoB tool but a 12-item modified version of the tool developed by the Cochrane Back Review Group. The four other studies were conducted by the same group of researchers. When our inter-rater reliability results for the RoB tool were compared to those of other studies, most kappa values for the RoB domains were similar, except for allocation concealment, incomplete data, selective reporting, and overall rating of the RoB tool. Our kappa values were much higher than those reported in previous studies (Table 4). We suggest a variety of reasons for these differences. Although we used the Cochrane Handbook guidelines for RoB assessments, we pre-defined specific decision rules to assess the individual domains of the tool. For example, the item of allocation concealment was scored low only when studies used central allocation (including telephone, web-based and centre controlled randomization) or when envelopes with three adequate safeguards were used (sequentially numbered, opaque, and sealed envelopes). If all three safeguards were not described, the item was scored as “unclear”. In addition to the Cochrane guidelines, the RoB item of incomplete data was rated “low” when intention to treat was conducted and the drop-out rate was less than or equal to 20%. When the drop-out rate was higher than 20%, the item was scored as “high” risk of bias since there is evidence that drop-out rates higher than 20% are likely to increase bias in treatment estimates [52], [53].

**Table 4:** Inter-rater reliability (kappa values) of the RoB tool reported in the scientific literature.

RoB Domains	Current Study	Hartling et al., 2011 [13]	Hartling et al., 2012 [14]	Hartling et al., 2009 [9]	Graham et al., 2012 [50]
Sequence generation	0.71	0.86	0.79	0.74	0.66
Allocation concealment	0.79	0.54	0.24	0.5	0.76
Blinding of participants and personnel	0.56	0.62	0.33	0.35	0.64
Blinding outcome assessment	0.54	0.62	0.33	0.35	0.5
Incomplete data	0.71	0.44	0.34	0.32	–
Selective reporting	0.50	0.40	0.27	0.13	–
Other bias	0.32	0.52	0.24	0.31	–
Overall rating	0.55	0.41	0.21	0.27	–
**Assessment characteristics**					
Type of trials	PT trials	Asthma trials	General health	Paediatric trials	Cervical/rehab trials
Number of trials	109	107	154	163	18
Trial evaluation specific to a single SR	No	Yes	No	No	NR
Number of trials used in pilot/training phase	10	??	5	5	NR
Number of reviewers	6	??	12	5	NR
Reviewers expertise	Physical therapy (2), methodology (6), public health (1), dentistry (1) and health related sciences (2). Doctorate (2), PhD candidate (1), Master level (1), undergraduate level (2)	NR	Doctorate (3); Master degree in health (8), epidemiology (1), undergraduate (1)	NR	Clinicians (physiotherapists, chiropractors, physicians), and statistician
Experience time conducting quality assessments	4 months – 10 years	NR	2–10 years	NR	5–50 years
Reviewers with formal training in SRs	3	NR	10	NR	NR
Outcomes used for RoB evaluation	Different outcome measures	Very specific outcomes	Different outcome measures	Different outcome measures	NR

RoB  =  Risk of Bias; SR  =  systematic review.

Similarly, we created a precise decision rule for the item of selective reporting, and identified a clear cut off to determine low, unclear and high RoB categories. It is likely that all of these decision rules may have increased the inter-reliability between the blinded external reviewers in the RoB assessments for these domains.

Final ratings of the RoB tool based on the Cochrane reviewers assessments indicated that almost 92% of trials included in the reviews had either high or unclear RoB; a proportion that is similar to those identified in other studies [10], [13]. As expressed by other researchers [13], the large number of trials classified as high or unclear RoB casts doubts about the discrimination power of the RoB tool to differentiate between studies with different levels of risk of bias that can explain variability of treatments effects across studies and inform accurately practice based on these assessments. **Thus, it is important to highlight that the overall assessment of the RoB may not be useful to determine quality of individual trials. We used the guidelines established by the Cochrane handbook to determine overall RoB. However, these criteria can be considered arbitrary and may not be appropriate**. **In addition**, the items included in the RoB may be insufficient to represent the construct of interest: “Risk of bias”. Other items not considered in this tool may need to be added to provide a more comprehensive evaluation. Some scales commonly used to evaluate the quality of research (e.g. the Jadad scale) use only a limited number of items (3) and have been criticized for their inability to distinguish among good and bad quality studies [54]. This may be a similar problem for the RoB, which may not include all important factors to evaluate the full construct of “risk of bias”. Empirical evidence supports the evaluation of randomization, allocation concealment and blinding of clinical trials, all of which are included in the RoB tool. While there is insufficient evidence to support other domains being included, other methodological factors could be important for evaluating RoB and could be considered for inclusion in the RoB tool after careful empirical evidence testing.

It is recommended that RoB assessments are made by multidisciplinary groups of reviewers, in which epidemiologists, methodologists, and clinicians with expertise in the content area of the review participate in the assessments. Our panel of reviewers had different levels of expertise, with two reviewers having at least 10 years of expertise in performing quality assessments and two of them with expertise in the area of the physical therapy. This might explain in part our higher levels of reliability compared to other studies.

When junior researchers are involved in RoB assessments, it is crucial that training in concepts and guidelines for assessing study bias is provided prior to the start of the review [4]. Training should be intense and monitored in each stage of the review. Previous studies have trained reviewers using an average of 5 trials per study. In contrast, we used 10 studies for training purposes and held regular meetings to discuss bias ratings of common papers. These factors may have helped to obtain acceptable levels of reliability between the external reviewer panel for most of the domains of the RoB tool.

### Limitations

This study restricted the analysis to a limited number of Cochrane systematic reviews in PT and therefore, the results might not reflect the inter-rater agreement of the RoB tool when applied to Cochrane reviews conducted in other areas of research, or to systematic reviews conducted out of the Cochrane Collaboration. Future studies should further assess potential differences in the inter-rater reliability of the RoB tool by comparing bias ratings of Cochrane reviews and non-Cochrane reviews versus those of independent panels of reviewers.

### Future directions

The reliability of RoB assessments applied to clinical trials in systematic reviews needs to be improved. The creation of an international database (a bias assessment bank) in which a qualified panel of experts (with extensive years of experience in trial methodology and critical appraisal of the scientific literature) contribute with independent RoB assessments of RCTs in a variety of clinical areas would be a promising step in that direction. Thus, researchers conducting systematic reviews and meta-epidemiological studies can use this data bank as a gold standard resource for RoB assessments. It is imperative that if an RoB assessment bank is created, contributors have the proper qualifications and experience to obtain less biased RoB assessments.

## Conclusions

As far of our knowledge, this study is the first to demonstrate that risk of bias assessments of RCTs using the RoB tool are not consistent across different research groups contrasting results from Cochrane reviewers with an independent external panel of reviewers. Poor agreement was not only demonstrated at the trial level but also at the meta-analysis level. These results have important implications for decision making since different recommendations can be reached depending on the group analyzing the evidence. Improved guidelines to apply the RoB tool and revisions to the tool for different health areas are needed. In addition, empirical evidence supporting additional items for the RoB tool needs to be developed. A call is made for the creation of a bank of RoB assessments of trial data, maintained by methodological and clinical experts that can be used as a reliable gold standard resource for RoB assessments. (4453 Words)

## Supporting Information

Appendix S1 **Search strategy to identify systematic review in physical therapy from the Cochrane Library of Systematic Reviews.**
(DOC)

Appendix S2 **Guidelines for evaluating the Risk of Bias in PT trials.**
(DOC)

Appendix S3 **Frequency of responses between Cochrane reviewers and the external panel of reviewers by RoB Domain.**
(DOC)
