# Supplementary material for: Poor Reliability between Cochrane Reviewers and Blinded External Reviewers When Applying the Cochrane Risk of Bias Tool in Physical Therapy Trials
Source: PLoS One. 2014 May 13;9(5):e96920. doi: 10.1371/journal.pone.0096920 (PMC4019638; doi:10.1371/journal.pone.0096920)
Supplement: Appendix S1 — Search strategy to identify systematic review in physical therapy from the Cochrane Library of Systematic Reviews. (DOC) [file pone.0096920.s001.doc]

**Appendix S1. Search strategy to identify systematic review in physical therapy from the Cochrane Library of Systematic Reviews**

| **#** | **Searches** | **Results** |
| --- | --- | --- |
| 1 | "physiotherapy":ti,ab,kw (Word variations have been searched) | 2560 |
| 2 | “Physical therapy” ti,ab,kw | 21184 |
| 3 | rehabilitation.mp. or exp "Physical and Rehabilitation Medicine" ti,ab,kw | 24400 |
| 4 | "exercise" ti,ab,kw | 35799 |
| 5 | “electrophysical agents” ti,ab,kw | 11 |
| 6 | “acupuncture” ti,ab,kw | 7410 |
| 7 | “Massage” ti,ab,kw | 1977 |
| 8 | “transcutaneous electrical stimulation” ti,ab,kw | 1078 |
| 9 | “interferential current ” ti,ab,kw | 99 |
| 10 | “ultrasound” ti,ab,kw | 9069 |
| 11 | “stretching” ti,ab,kw | 2044 |
| 12 | “chest therapy” ti,ab,kw | 6332 |
| 13 | “pulmonary rehabilitation” ti,ab,kw | 1558 |
| 14 | “manipulative therapy” ti,ab,kw | 579 |
| 15 | “exp Musculoskeletal Manipulations/ or musculoskeletal therapy.mp. | 2391 |
| 16 | “manual ther*.mp. | 5318 |
| 17 | “mobilization” ti,ab,kw |  |
| 18 | exp Exercise/ or exp Exercise Movement Techniques/ or exercise.mp. or exp Exercise Therapy/ | 679 |
| 19 | physiotherap*.mp. | 5483 |
| 20 | physical therap*.mp. | 22576 |
| 21 | #1 or #2 or #3 or #4 or #5 or #6 or #7 or #8 or #9 or #10 or #11 or #12 or #13 or #14 or #15 or #16 or #17 or #18 or #19 or #20 | 92581 |
| 22 | Systematic reviews | 38259 |
| 23 | # 21 AND #22 | 10265 |
| 36 | Limit to Cochrane reviews | 3901 |
