# Supplementary material for: Poor Reliability between Cochrane Reviewers and Blinded External Reviewers When Applying the Cochrane Risk of Bias Tool in Physical Therapy Trials
Source: PLoS One. 2014 May 13;9(5):e96920. doi: 10.1371/journal.pone.0096920 (PMC4019638; doi:10.1371/journal.pone.0096920)
Supplement: Appendix S2 — Guidelines for evaluating the Risk of Bias in PT trials. (DOC) [file pone.0096920.s002.doc]

**Appendix S2. Guidelines for evaluating the Risk of Bias in PT trials.**

Most of the guidelines were taken from the Cochrane Handbook for Systematic Reviews of Interventions. However, we created some decision rules to help with consistency in assessments and are intended to supplement the Cochrane guidelines.

| **RANDOM SEQUENCE GENERATION**  **Selection bias (biased allocation to interventions) due to inadequate generation of a randomised sequence.** | |
| --- | --- |
| Criteria for a judgement of  **‘Low risk’ of bias.** | The investigators describe a random component in the sequence generation process such as:   - Referring to a random number table; - Using a computer random number generator; - Coin tossing; - Shuffling cards or envelopes; - Throwing dice; - Drawing of lots; - Minimization*.     *Minimization may be implemented without a random element, and this is considered to be equivalent to being random. |
| Criteria for the judgement of ‘**High risk’** of bias. | The investigators describe a non-random component in the sequence generation process. Usually, the description would involve some systematic, non-random approach, for example:   - Sequence generated by odd or even date of birth; - Sequence generated by some rule based on date (or day) of admission; - Sequence generated by some rule based on hospital or clinic record number.   Other non-random approaches happen much less frequently than the systematic approaches mentioned above and tend to be obvious.  They usually involve judgement or some method of non-random categorization of participants, for example:   - Allocation by judgement of the clinician; - Allocation by preference of the participant; - Allocation based on the results of a laboratory test or a series of tests; - Allocation by availability of the intervention. |
| Criteria for the judgement of ‘Unclear risk’ of bias. | Insufficient information about the sequence generation process to permit judgement of ‘Low risk’ or ‘High risk’. |
| **ALLOCATION CONCEALMENT**  Selection bias (biased allocation to interventions) due to inadequate concealment of allocations prior to assignment. | |
| Criteria for a judgement of ‘Low risk’ of bias. | Participants and investigators enrolling participants could not foresee assignment because one of the following, or an equivalent method, was used to conceal allocation:   - Central allocation (including telephone, web-based and pharmacy-controlled randomization); - Sequentially numbered drug containers of identical appearance; - Sequentially numbered, opaque, sealed envelopes (3 characteristics need to be present) |
| Criteria for the judgement of ‘High risk’ of bias. | Participants or investigators enrolling participants could possibly foresee assignments and thus introduce selection bias, such as allocation based on:   - Using an open random allocation schedule (e.g. a list of random numbers); - Assignment envelopes were used without appropriate safeguards (e.g. if envelopes were unsealed or non­opaque or not sequentially numbered); - Alternation or rotation; - Date of birth; - Case record number; - Any other explicitly unconcealed procedure. |
| Criteria for the judgement of ‘Unclear risk’ of bias. | Insufficient information to permit judgement of ‘Low risk’ or ‘High risk’. This is usually the case if the method of concealment is not described or not described in sufficient detail to allow a definite judgement – for example if the use of assignment envelopes is described, but it remains unclear whether envelopes were sequentially numbered, opaque and sealed. **(Not all of 3 characteristics are present).** |
| **BLINDING OF PARTICIPANTS AND PERSONNEL (Main Outcome)**   - Blinding of participants/patients is a **“MUST”** when outcomes are subjective or self-reported. - When Outcomes are measured by an assessor, then assessors should be blinded to group allocation. - When Outcomes are automated [database]( there is no assessor involved) then, blinding of participants or assessors is NOT an issue   Performance bias due to knowledge of the allocated interventions by participants and personnel during the study. | |
| Criteria for a judgement of ‘Low risk’ of bias. | Any one of the following:   - No blinding or incomplete blinding, but the review authors judge that the outcome is not likely to be influenced by lack of blinding (Automated outcome or administrative) - Blinding of participants and key study personnel ensured, and unlikely that the blinding could have been broken - Objectives automatized outcomes coming from databases or hospital register office. |
| Criteria for the judgement of ‘High risk’ of bias. | Any one of the following:   - No blinding or incomplete blinding, and the outcome is likely to be influenced by lack of blinding; - Blinding of key study participants and personnel attempted, but likely that the blinding could have been broken, and the outcome is likely to be influenced by lack of blinding. |
| Criteria for the judgement of ‘Unclear risk’ of bias. | Any one of the following:   - Insufficient information to permit judgement of ‘Low risk’ or ‘High risk’; - The study did not address the issue of blinding. |
| **BLINDING OF OUTCOME ASSESSMENT**  Detection bias due to knowledge of the allocated interventions by outcome assessors. | |
| Criteria for a judgement of ‘Low risk’ of bias. | Any one of the following:   - No blinding of outcome assessment, but the review authors judge that the outcome measurement is not likely to be influenced by lack of blinding; - Blinding of outcome assessment ensured, and unlikely that the blinding could have been broken. |
| Criteria for the judgement of ‘High risk’ of bias. | Any one of the following:   - No blinding of outcome assessment, and the outcome measurement is likely to be influenced by lack of blinding; - Blinding of outcome assessment, but likely that the blinding could have been broken and the outcome measurement is likely to be influenced by lack of blinding. |
| Criteria for the judgement of ‘Unclear risk’ of bias. | Any one of the following:   - Insufficient information to permit judgement of ‘Low risk’ or ‘High risk’; - The study did not address the issue of blinding. |
| **INCOMPLETE OUTCOME DATA**  Attrition bias due to amount, nature or handling of incomplete outcome data. | |
| Criteria for a judgement of ‘Low Low risk’ of bias. | Any one of the following:   - No missing outcome data (If all patients were accounted for in the analysis) - Reasons for missing outcome data unlikely to be related to true outcome (for survival data, censoring unlikely to be introducing bias); - Missing outcome data balanced in numbers across intervention groups, with similar reasons for missing data across groups; - For dichotomous outcome data, the proportion of missing outcomes compared with observed event risk not enough to have a clinically relevant impact on the intervention effect estimate; - For continuous outcome data, plausible effect size (difference in means or standardized difference in means) among missing outcomes not enough to have a clinically relevant impact on observed effect size; - Missing data have been imputed using appropriate methods. - If authors claimed that an intention-to-treat analysis was performed, raters should confirm that all patients entered were accounted for in the analysis (i.e., do not assume that a true intention-to-treat analysis was done). - If the numbers and reasons for withdrawal/drop-out were described and comparable across groups **and the authors performed an Intention to treat with ≤ 20% drop outs, then score low risk of bias** - If the numbers and reasons for withdrawal/drop-out were described and comparable across groups **but they did not perform an ITT and the drop out rate was less or equal than 10% drop outs, then score low risk of bias** |
| Criteria for the judgement of ‘High risk’ of bias. | Any one of the following:   - Reason for missing outcome data likely to be related to true outcome, with either imbalance in numbers or reasons for missing data across intervention groups; - For dichotomous outcome data, the proportion of missing outcomes compared with observed event risk enough to induce clinically relevant bias in intervention effect estimate; - For continuous outcome data, plausible effect size (difference in means or standardized difference in means) among missing outcomes enough to induce clinically relevant bias in observed effect size; - ‘As-treated’ analysis done with substantial departure of the intervention received from that assigned at randomization; - Potentially inappropriate application of simple imputation. - **No Intention to treat (ITT) or ITT performed with > 20% drop outs** |
| Criteria for the judgement of ‘Unclear risk’ of bias. | Any one of the following:   - Insufficient reporting of attrition/exclusions to permit judgement of ‘Low risk’ or ‘High risk’ (e.g. number randomized not stated, no reasons for missing data provided); - The study did not address this outcome. - **No Intention to treat (ITT) but >10% and ≤ 20% drop outs** |
| **SELECTIVE REPORTING (Are outcomes reported in methods and results?)**  Reporting bias due to selective outcome reporting. | |
| Criteria for a judgement of ‘Low risk’ of bias. | Any of the following:   - The study protocol is available and all of the study’s pre-specified (primary and secondary) outcomes that are of interest in the review have been reported in the pre-specified way; - The study protocol is not available but it is clear that the published reports include all expected outcomes, including those that were pre-specified (convincing text of this nature may be uncommon). - **Since we are NOT searching for protocols, OUTCOMES reported in methods section need to match those reported in results section** - **If 0-30% of the secondary outcomes are not reported score low risk.** - **Main outcome has to be included in both methods and results** |
| Criteria for the judgement of ‘High risk’ of bias. | Any one of the following:   - Not all of the study’s pre-specified primary outcomes have been reported; - One or more primary outcomes is reported using measurements, analysis methods or subsets of the data (e.g. subscales) that were not pre-specified; - One or more reported primary outcomes were not pre-specified (unless clear justification for their reporting is provided, such as an unexpected adverse effect); - One or more outcomes of interest in the review are reported incompletely so that they cannot be entered in a meta-analysis; - The study report fails to include results for a key outcome that would be expected to have been reported for such a study. - **≥ 70% of secondary outcomes were UNREPORTED ( combining methods or results sections)** - **If main Outcome was not reported in the study score as high risk of bias** |
| Criteria for the judgement of ‘Unclear risk’ of bias. | - **Insufficient information to permit judgement of ‘Low risk’ or ‘High risk’. It is likely that the majority of studies will fall into this category.** - **If between 31%-69% of secondary outcomes are UNREPORTED ( combining methods or results sections) score as unclear risk of bias** |
| **OTHER BIAS**  Bias due to problems not covered elsewhere in the table. | |
| Criteria for a judgement of ‘Low risk’ of bias. | The study appears to be free of other sources of bias. |
| Criteria for the judgement of ‘High risk’ of bias. | There is at least one important risk of bias. For example, the study:   - Had a potential source of bias related to the specific study design used; or - Has been claimed to have been fraudulent; or - Had some other problem. - **If the study has baseline imbalances regarding demographic factors, duration and severity of complaints, and value of main outcome measure(s).**[**^1^**](#_ENREF_1) - **Imbalances in co-interventions: if the co-interventions were imbalances between groups or they were NOT similar between groups**[**^1^**](#_ENREF_1) - **Compliance with treatment was not acceptable (very poor adherence with actual treatment: e.g. exercises performed) based on the reported intensity, duration, number and frequency of sessions for both the index intervention and control intervention(s). For example, physiotherapy treatment is usually administered over several sessions; therefore it is necessary to assess how many sessions each patient attended. For single session interventions this item is irrelevant.** [^1^](#_ENREF_1) |
| Criteria for the judgement of ‘Unclear risk’ of bias. | There may be a risk of bias, but there is either:   - Insufficient information to assess whether an important risk of bias exists; or - Insufficient rationale or evidence that an identified problem will introduce bias. |

**Total Scoring for RoB tool:**

**If any High Risk = High Risk of Bias**

**If any Unclear and NO high Risk = Unclear Risk of Bias**

**If all of the items are Low risk= Low Risk of Bias**

***The statements in bold were added by our study team**

**1.** Furlan AD, Pennick V, Bombardier C, Van Tulder M. 2009 Updated method guidelines for systematic reviews in the cochrane back review group. *Spine.* // 2009;34(18):1929-1941.
