# Supplementary material for: Poor Reliability between Cochrane Reviewers and Blinded External Reviewers When Applying the Cochrane Risk of Bias Tool in Physical Therapy Trials
Source: PLoS One. 2014 May 13;9(5):e96920. doi: 10.1371/journal.pone.0096920 (PMC4019638; doi:10.1371/journal.pone.0096920)
Supplement: Appendix S3 — Frequency of responses between Cochrane reviewers and the external panel of reviewers by RoB Domain. (DOC) [file pone.0096920.s003.doc]

**Appendix S3. Frequency of responses between Cochrane reviewers and the external panel of reviewers by RoB Domain**

|  | **External Panel of Reviewers** | **Cochrane Reviewers** | | | |
| --- | --- | --- | --- | --- | --- |
| **RoB Domain** |  | **Low** | **Unclear** | **High** | **Total** |
| **Sequence Generation** | **Low** | **39** | **4** | **0** | **43** |
|  | **Unclear** | **14** | **48** | **4** | **66** |
|  | **Total** | **53** | **52** | **4** | **109** |
|  | | | | | |
| **Allocation Concealment** | **Low** | **20** | **3** | **0** | **23** |
|  | **Unclear** | **31** | **45** | **9** | **85** |
|  | **Total** | **51** | **48** | **9** | **108** |
|  | | | | | |
| **Blinding participants and personnel** | **Low** | **21** | **7** | **11** | **39** |
|  | **Unclear** | **10** | **15** | **18** | **43** |
|  | **High** | **2** | **1** | **1** | **4** |
|  | **Total** | **33** | **23** | **30** | **86** |
|  | | | | | |
| **Blinding of outcome assessors** | **Low** | **31** | **2** | **4** | **37** |
|  | **Unclear** | **12** | **22** | **16** | **50** |
|  | **High** | **3** | **2** | **5** | **10** |
|  | **Total** | **46** | **26** | **25** | **97** |
|  | | | | | |
| **Incomplete Outcome assessment** | **Low** | **40** | **7** | **6** | **53** |
|  | **Unclear** | **16** | **6** | **8** | **30** |
|  | **High** | **6** | **1** | **6** | **13** |
|  | **Total** | **62** | **14** | **20** | **96** |
|  | | | | | |
| **Selective reporting** | **Low** | **41** | **25** | **4** | **70** |
|  | **Unclear** | **7** | **5** | **1** | **13** |
|  | **High** | **1** | **1** | **2** | **4** |
|  | **Total** | **49** | **31** | **7** | **87** |
|  | | | | | |
| **Other bias** | **Low** | **3** | **1** | **4** | **8** |
|  | **Unclear** | **34** | **18** | **17** | **69** |
|  | **High** | **0** | **1** | **0** | **1** |
|  | **Total** | **37** | **20** | **21** | **78** |
|  | | | | | |
| **Overall RoB** | **Low** | **0** | **0** | **3** | **3** |
|  | **Unclear** | **4** | **26** | **45** | **75** |
|  | **High** | **5** | **8** | **18** | **31** |
|  | **Total** | **9** | **33** | **66** | **109** |
|  |  |  |  |  |  |
| **RoB at the Meta-analysis level** | **Low** | **0** | **0** | **0** | **0** |
|  | **Unclear** | **0** | **3** | **10** | **13** |
|  | **High** | **1** | **2** | **1** | **4** |
|  | **Total** | **1** | **5** | **11** | **17** |
